# Post-Pandemic Trends of 24-Hour Movement Behaviours and Mental Health Outcomes in Youth

**DOI:** 10.3390/children13101407

**Published:** 2026-10-14

**Authors:** Tiago D. Ribeiro, Miguel Peralta, Adilson Marques, Nuno Neto Rodrigues, Margarida Gaspar de Matos

**Affiliations:** 1 Centro Interdisciplinar de Performance Humana (CIPER), Faculdade de Motricidade Humana, Universidade de Lisboa, 1499-002 Lisbon, Portugal; tiagoribeiro@fmh.ulisboa.pt (T.D.R.); mperalta@fmh.ulisboa.pt (M.P.); amarques@fmh.ulisboa.pt (A.M.); 2 Instituto de Saúde Ambiental (ISAMB), Faculdade de Medicina, Universidade de Lisboa, 1649-004 Lisbon, Portugal; 3 Núcleo de Investigación en Ciencias del Movimiento, Universidad Arturo Prat, Iquique 1110939, Chile; 4 Direção-Geral de Estatísticas da Educação e Ciência, Avenida 24 de Julho 134, 1200-774 Lisbon, Portugal; 5 Faculdade de Ciências Humanas, Universidade Católica Portuguesa, 1649-023 Lisbon, Portugal

**Keywords:** anxiety, depression, physical activity, screen time, sleep, stress

## Abstract

**Highlights:**

**What are the main findings?**

- From 2022 to 2024, adherence to physical activity and screen-time guidelines increased, yet only 1.9% of Portuguese youth met all three 24-h movement guidelines in 2024.
- Meeting physical activity, screen-time and sleep guidelines was associated with the lowest odds of depression, anxiety and stress.

**What are the implications of the main findings?**

- Youth mental health interventions may benefit from combining sleep support, recreational screen-use management and opportunities for daily physical activity.
- These findings may inform coordinated public health initiatives across schools, families and communities to support adolescent mental health at the population level.

**Abstract:**

**Background/Objectives**: The COVID-19 pandemic disrupted youth movement behaviours and mental health, but evidence on post-pandemic trends remains limited. Therefore, this study examined differences in compliance with the 24-h movement behaviour guidelines and their association with mental health outcomes among Portuguese children and adolescents between 2022 and 2024. **Methods**: This repeated cross-sectional study included two independent samples of Portuguese children and adolescents from the 2022 and 2024 waves of the Health Behaviour in School-aged Children study (2022: n = 2775; 2024: n = 2381). The 24-h movement behaviours (physical activity, sleep and screen time) and mental health outcomes, assessed by Depression Anxiety Stress Scales—21 (DASS-21), were self-reported. Prevalences of attaining the 24-h movement guidelines and DASS-21 categories were calculated and compared between 2022 and 2024 using chi-square tests. Adjusted logistic regression models were used to examine the associations between movement behaviour compliance and mental health outcomes. **Results**: From 2022 to 2024, the proportion meeting physical activity (7.5% to 13.7%; *p* < 0.001) and screen-time (12.8% to 28.2%; *p* < 0.001) guidelines increased, while adherence to sleep recommendations did not differ significantly (42.4% to 41.2%; *p* = 0.365). The proportion meeting two or more guidelines was also higher in 2024 (9.5% to 20.1%; *p* < 0.001). The prevalence of mild-to-extremely severe symptoms was lower in 2024 than in 2022 for depression (34.2% to 43.9%; *p* < 0.001), anxiety (38.1% to 43.2%; *p* = 0.001) and stress (23.6% to 31.0%; *p* < 0.001). Meeting screen-time and sleep guidelines was associated with at least 31% lower odds of having depressive, anxiety and stress symptoms (*p* < 0.001). **Conclusions**: More favourable prevalence estimates for movement behaviours and mental health outcomes were observed in 2024 than in 2022. Compliance with screen-time and sleep guidelines was associated with lower odds of depressive, anxiety and stress symptoms.

## 1. Introduction

The COVID-19 pandemic significantly disrupted the daily routines of children and adolescents worldwide, leading to declines in physical activity levels and increases in sedentary behaviours and sleep duration [1]. These changes were largely driven by prolonged school closures, restricted access to outdoor spaces, and increased reliance on digital technologies for education and socialisation. Beyond 24-h movement behaviour disruptions, the pandemic also had an impact on the mental health of young people, with several investigations documenting increased rates of depression, anxiety, and stress symptoms among adolescents during the pandemic period [2]. Given the implications of mental health problems for young people’s well-being and development, continued monitoring beyond the acute phase of the pandemic is important to determine whether the elevated symptom prevalence observed during this period persists and to inform prevention and support strategies.

Mental health outcomes are closely linked to the 24-h movement behaviours. The 24-h movement behaviours refer to the integrated pattern of physical activity, sedentary behaviour (including screen time), and sleep across the whole day [3]. This paradigm recognises that these behaviours are interdependent and mutually influential, rather than isolated components of lifestyle. Emerging research supports the idea that physical activity, limited screen time, and adequate sleep are jointly associated with better mental health among young people [4,5,6].

Several pre-pandemic studies have documented low compliance with all three 24-h movement guidelines in South Korea (2013–2018), the United States (2007–2016), and Australia (2009–2018) [7,8,9]. A Spanish study comparing pre-pandemic data collected in March–June 2018 with data collected in February–March 2021, approximately 1 year after the onset of the pandemic, found lower compliance with all three guidelines in 2021 [10]. This study provides evidence from the peri-pandemic period, whereas evidence on subsequent post-pandemic patterns remains limited.

In Portugal, nationally representative data on post-pandemic trends in adolescents’ movement behaviours and mental health are still limited, despite the availability of large-scale surveillance systems such as the Health Behaviour in School-aged Children (HBSC) study. This represents an important gap, as population-level patterns may vary across national contexts. Focusing on the post-pandemic period complements comparisons with pre-pandemic baselines by describing more recent patterns in movement behaviours and mental health. Repeated cross-sectional surveys can identify differences between survey waves, although they cannot establish individual recovery trajectories or attribute these differences to the pandemic.

Despite growing recognition of the importance of the 24-h movement framework, little is known about post-pandemic differences in guideline compliance and mental health outcomes, and their associations during this period. Most existing studies are either cross-sectional, focus on single behaviours, or do not include mental health outcomes simultaneously. Therefore, there is a clear gap in understanding temporal differences in these indicators and their cross-sectional associations after the pandemic. Thus, this study aims to compare compliance with the 24-h movement behaviour guidelines and the prevalence of depression, anxiety, and stress symptoms between independent samples of Portuguese children and adolescents surveyed in 2022 and 2024, and to examine associations between guideline compliance and these symptoms. We hypothesised that guideline compliance would remain similar or be slightly higher in 2024 than in 2022, while the prevalence of depression, anxiety, and stress symptoms would remain similar or be slightly lower. We also hypothesised that meeting a greater number of movement guidelines would be associated with lower odds of these symptoms.

## 2. Materials and Methods

### 2.1. Study Design

This is an observational analytical study with a repeated cross-sectional design, using two independently selected samples from the 2022 and 2024 waves of the Health Behaviour in School-aged Children (HBSC) study in Portugal.

### 2.2. Procedures and Participants

The HBSC is a cross-national survey conducted in collaboration with the World Health Organisation (WHO) Regional Office for Europe. The study uses a nationally representative sample of children and adolescents attending public and private schools across mainland Portugal. Schools were randomly selected through a stratified sampling process by NUTS III (Nomenclature of Territorial Units for Statistics) regions. Eligible participants were students from middle to high school (5th through 12th grade). These grades generally correspond to ages 10–18 years, although younger and older students may attend them. The dataset available for this analysis contained 4444 records from 2022 and 3073 records from 2024 before the application of eligibility and missing-data criteria. For this study, participants without data on age, grade, school social support tier, movement behaviours (physical activity, sedentary behaviour, sleep) and any item of the Depression Anxiety Stress Scales—21 (DASS-21) were excluded. The final analytical sample included 2775 participants from 2022 and 2381 from 2024. Sample-size adequacy was assessed using the noncentral chi-square distribution for comparison of the five DASS-21 severity categories between survey years. Assuming a small effect size (Cohen’s w = 0.10), α = 0.05 and 80% power, the required total sample was 1194 participants (4 degrees of freedom), below the available sample of 5156. This calculation assumes independent observations, does not account for clustering or weighting, and does not establish adequate precision for subgroup regression estimates. Participation was anonymous and voluntary, with informed consent obtained from legal guardians for participants younger than 18 years and directly from participants aged 18 years or older. Data were collected through an anonymous, self-completed online questionnaire administered during school hours. Further methodological information is available in the international HBSC study protocol [11].

### 2.3. Measures

#### 2.3.1. Movement Behaviours

Movement behaviours were self-reported by children and adolescents. Physical activity was assessed using the question, “During the last 7 days, on how many days did you engage in physical activity for a total of at least 60 min?”. Response options ranged from 0 to 7 days. Sleep duration was assessed using the question, “In general, how many hours do you sleep each night?”. Screen time was measured by asking participants, “In general, how many hours do you spend each day in front of a screen (television, mobile phone, computer or tablet) for recreational activities?”. For both sleep duration and screen time, response options ranged from 0 to “10 or more h”, in one-h increments. Both questions assessed usual duration without specifying a fixed recall period.

These behaviours were categorised using criteria based on the Canadian 24-Hour Movement Guidelines [3]. Participants were considered to meet the physical activity recommendation if they engaged in at least 60 min of moderate-to-vigorous physical activity (MVPA) every day. Sleep was considered adequate when children aged 9–13 years reported 9 or more h per night, adolescents aged 14–17 years reported 8–10 h, and those aged 18–20 years reported 7–9 h. Compliance with screen-time recommendations was defined as reporting 2 h or less per day of recreational screen use. A combined indicator, referred to as 24-h movement behaviours, was created by summing the number of individual recommendations met, resulting in a score ranging from 0 to 3.

#### 2.3.2. Mental Health Outcomes

Mental health outcomes were assessed using the Portuguese version of the DASS-21, a validated self-report instrument designed to measure symptoms of depression, anxiety, and stress [12,13]. The questionnaire consists of 21 items divided equally among three subscales referring to the previous week. Each item is rated on a 4-point Likert scale, ranging from 0 (“did not apply to me at all”) to 3 (“applied to me most of the time”). In line with validated cut-off scores [12,14], seven item scores were summed within each subscale and multiplied by two. Scores of ≥10 for depression, ≥8 for anxiety and ≥15 for stress were classified as indicating at least mild symptoms. For this study, each outcome was dichotomised into “mild to extremely severe symptoms” versus “normal” to distinguish participants with scores within the normal range from those reporting at least mild symptom severity.

### 2.4. Data Analysis

Descriptive statistics were calculated for all variables included in the study. The chi-square test was used to compare the proportions of psychological symptoms (depression, anxiety, and stress) and compliance with each movement behaviour (physical activity, screen time, and sleep) between 2022 and 2024 in the overall sample and separately by sex. Absolute prevalence differences between 2024 and 2022 were expressed in percentage points, with 95% confidence intervals calculated using the normal approximation for two independent proportions. To assess the association between movement behaviours and psychological symptoms, binary logistic regression models were conducted for each outcome (depression, anxiety, and stress), using pooled data from both survey waves. Separate models examined individual movement behaviours (physical activity, screen time, and sleep) and the number of recommendations met (0 to 3). The models included 24-h movement behaviours (physical activity, screen time, and sleep) as well as a combined variable reflecting the number of recommendations met (0 to 3). All models were adjusted for survey year, sex, age, grade, and school social support. Models examining individual movement behaviours were additionally adjusted for the other movement behaviours. Results are presented as odds ratios (OR) with 95% confidence intervals (CI). All analyses were performed using IBM SPSS Statistics version 28 (IBM Corp., Armonk, NY, USA), and a significance level of 0.05 was used. To assess the robustness of the findings, sensitivity analyses were conducted using continuous DASS-21 depression, anxiety, and stress scores as outcomes in adjusted linear regression models.

## 3. Results

A total of 5156 Portuguese children and adolescents (2478 boys and 2678 girls) participated in the study, including 2775 in 2022 and 2381 in 2024. The mean age was 14.2 ± 2.2 years. Overall, participants were distributed across all school grades from 5th to 12th. Most participants (58.2%) did not receive school social support, which is typically allocated to students from lower-income households. Sociodemographic characteristics of the participants, stratified by survey year and sex, are detailed in Table 1.

Movement behaviour prevalence estimates for 2022 and 2024 are presented in Table 2. The proportion meeting physical activity guidelines increased from 7.5% to 13.7% (difference: 6.2 percentage points; 95% CI: 4.5, 7.9; *p* < 0.001). Screen-time compliance rose from 12.8% to 28.2% (difference: 15.4 percentage points; 95% CI: 13.2, 17.6; *p* < 0.001), while adherence to sleep recommendations did not differ significantly between years (42.4% versus 41.2%; difference: −1.3 percentage points; 95% CI: −4.0, 1.4; *p* = 0.365). The proportion meeting none of the three 24-h movement behaviour guidelines decreased from 47.8% to 38.9% (difference: −8.9 percentage points; 95% CI: −11.6, −6.2), and the proportion meeting two or more increased from 9.5% to 20.1% (difference: 10.6 percentage points; 95% CI: 8.7, 12.5). Boys consistently reported more favourable movement behaviour profiles than girls.

Mental health prevalence estimates were more favourable in 2024 than in 2022. The proportion of participants with normal scores on the DASS-21 depression subscale increased from 56.1% in 2022 to 65.8% in 2024 (difference: 9.7 percentage points; 95% CI: 7.0, 12.4), while severe or extremely severe depression decreased from 14.8% to 10.2% (difference: −4.6 percentage points; 95% CI: −6.4, −2.8). Similar trends were observed for anxiety and stress: normal scores in anxiety rose from 56.8% to 61.9% (difference: 5.0 percentage points; 95% CI: 2.4, 7.7), and normal scores in stress from 69.0% to 76.4% (difference: 7.4 percentage points; 95% CI: 5.0, 9.8). The distributions across severity categories differed significantly between years for depression (*p* < 0.001), anxiety (*p* = 0.001) and stress (*p* < 0.001). Girls consistently reported higher prevalences of psychological symptoms. Within each sex, comparisons between years showed significant differences in severity distributions for all three subscales among girls (all *p* < 0.001), but not among boys (depression: *p* = 0.136; anxiety: *p* = 0.454; stress: *p* = 0.787).

In the pooled sample, logistic regression models showed that meeting screen-time and sleep guidelines was significantly associated with lower odds of experiencing mild to extremely severe symptoms of depression, anxiety, and stress (see Table 3). For instance, those meeting sleep guidelines had 48% lower odds of depressive symptoms (OR = 0.52, 95% CI: 0.46, 0.59). Meeting the physical activity guideline was not significantly associated with any of the DASS-21 outcomes. Participants meeting all three movement recommendations had lower odds of mild to extremely severe symptoms across all three subscales compared to those meeting none. Sensitivity analyses using continuous DASS-21 scores produced results consistent with the primary analyses. Compliance with screen-time and sleep recommendations remained significantly associated with lower depression, anxiety, and stress scores (B ranging from −0.81 to −1.68 and from −0.86 to −1.50, respectively), whereas compliance with physical activity recommendations was not significantly associated with any outcome.

## 4. Discussion

To our knowledge, this is among the first studies examining post-pandemic trends in Portuguese youth’s 24-h movement behaviours (physical activity, screen time, and sleep) and mental health outcomes (depression, anxiety, stress). More favourable prevalence estimates for movement behaviour compliance were observed in 2024 than in 2022, particularly in the proportion of children and adolescents meeting physical activity (from 7.5% to 13.7%) and screen-time guidelines (12.8% to 28.2%), while adequate sleep duration remained relatively unchanged. Concurrently, mental health prevalence estimates were more favourable in 2024. Girls reported higher prevalences of psychological symptoms than boys in both survey waves. These findings describe differences between independent samples and do not demonstrate individual recovery.

Several factors could help explain the differences observed between 2022 and 2024. Changes in school routines, opportunities for sport and face-to-face socialising, and access to mental health support are possible explanations, but these factors were not assessed in this study. Our findings are consistent with longitudinal evidence from Italy, Spain and Portugal reporting reductions in psychological symptoms between the initial pandemic period and 2023 [15]. However, differences in sample composition, measurement conditions, secular trends and other unmeasured contextual factors could also contribute to the observed differences.

In international terms, however, compliance with the 24-h movement guidelines remains low. Even with the more favourable estimates, only a minority of Portuguese adolescents met two or more recommendations in 2024. This is consistent with evidence from other countries showing persistently low adherence to integrated movement behaviour guidelines before the pandemic [7,9]. These findings highlight that substantial gaps remain in achieving healthy daily behaviour patterns among adolescents.

It is also important to note the sex differences in symptom prevalence. In both survey waves, depression, anxiety and stress symptoms were more prevalent among girls than boys. Boys, in contrast, exhibited lower baseline symptom levels and did not report statistically significant changes over the two years, potentially due to differing coping strategies or patterns of physical and social activity. Previous research from Iceland reported lower depressive symptom levels in 2023 than in 2021, although symptoms remained above pre-pandemic levels [16]. From a public health standpoint, the persistently higher symptom prevalence among girls supports the need for continued monitoring and targeted interventions to address the disparities.

The associations between attaining the 24-h movement behaviour guidelines and mental health outcomes were also explored. In the pooled sample, meeting the recommended screen-time and sleep duration guidelines was associated with substantially lower odds of reporting depression, anxiety, or stress symptoms. The significant association between sufficient sleep and lower odds of mental health symptoms aligns with a growing body of evidence underscoring the crucial role of sleep in adolescent well-being [17]. This association remained after adjustment for physical activity and screen time. However, the present study assessed sleep duration, not sleep quality, continuity or insomnia symptoms. These dimensions should be distinguished, and the associations between sleep and mental health may be bidirectional. Also, these findings align with the literature linking excessive screen time with depression and anxiety symptoms in children and adolescents, potentially through mechanisms such as reduced sleep quality, displacement of in-person social interaction and exposure to harmful online content [18]. These are possible explanations rather than mechanisms tested in this study, as the screen-time measure did not distinguish content or context of use. Notably, the combination of low screen time and adequate sleep had one of the strongest relationships with mental health in prior studies [19], and our results support the importance of this combination. However, specific combinations were not examined, so our findings cannot establish the association of this particular combination with mental health.

Notably, compliance with the physical activity guideline was not significantly linked to lower odds of symptoms in adjusted models. However, adolescents meeting all three movement behaviour recommendations had lower odds of depression, anxiety and stress symptoms than those meeting none. The physical activity recommendation (≥60 min of moderate-to-vigorous physical activity daily) was a threshold that few children and adolescents met. However, this binary classification does not capture differences in activity below the threshold and cannot establish whether a dose–response relationship exists. Prior recent research includes intervention evidence suggesting beneficial effects of physical activity on depression and anxiety symptoms, although the quality of evidence remains limited [20]. Therefore, our results do not necessarily contradict the mental health benefits of physical activity; the present observational comparison of guideline compliance addresses a different question from studies evaluating the effects of physical activity interventions. This integrated perspective is consistent with the concept of the 24-h movement framework, whereby the interconnectedness of behaviours across the whole day is important for health.

### 4.1. Practical Implications

The associations between meeting multiple movement behaviour guidelines and lower odds of psychological symptoms suggest that an integrated approach to adolescent health interventions warrants further evaluation. Our findings do not establish whether modifying these behaviours improves mental health or whether interventions targeting multiple behaviours are more effective than those targeting a single behaviour. Schools, parents, and healthcare providers could aim to create supportive environments that facilitate all these behaviours together. For instance, school programs could combine physical activity opportunities (daily exercise sessions or active breaks) with sleep education (teaching good sleep hygiene and the importance of adequate rest) and digital awareness (helping students manage and critically reflect on their screen use). At home, families may establish a routine that helps children and adolescents to meet the guidelines [21].

Screen-time duration alone does not capture the potential relevance of content and context. Social media use and other online activities may involve exposure to cyberbullying, body image pressures, or disturbing news, while screen use may displace sleep time or affect sleep quality [22]. These pathways were not assessed in the present study. Adequate sleep, on the other hand, is fundamental for emotional regulation, learning, and stress resilience [23]. Therefore, sleep habits and screen use may be considered potential intervention targets, but their effects on depression, anxiety and stress require prospective and experimental evaluation.

School-based delivery is illustrated by an intervention combining physical activity and nutrition education among 12th-grade students during the COVID-19 pandemic, which reported favourable changes in physical activity and eating habits [24]. Although that study did not assess mental health outcomes, it provides an example of implementing multicomponent health education in schools.

### 4.2. Limitations and Strengths

This study has some limitations that need to be acknowledged. The repeated cross-sectional design, based on two independent samples, allows comparisons between survey waves but cannot establish within-person changes or recovery trajectories. The cross-sectional associations within the pooled data also preclude conclusions about temporal ordering or causality, and reverse causation is possible. Differences in sample composition and residual confounding may have influenced the findings. Moreover, all data on movement behaviours and psychological symptoms were self-reported, which introduces potential recall and social desirability biases. The use of binary movement criteria also reduced information on behavioural variation. Applying the same physical activity and screen-time thresholds across ages facilitated comparisons but may not reflect age-specific recommendations for participants aged 18–20 years. DASS-21 categories indicate symptom severity rather than clinical diagnoses, and dichotomisation combined participants with different levels of severity. The applicability of the Portuguese DASS-21 validation to the youngest participants also requires caution. There were also instances of missing data among the variables included, which may have affected the representativeness of the sample of Portuguese school-attending youth. Furthermore, the small number of participants meeting all three recommendations limited the precision of estimates for this group. Despite these limitations, the study offers several strengths, including a large sample size, standardised data collection procedures, and the capacity to assess multiple health behaviours and outcomes concurrently. These findings provide timely and valuable insights into adolescent health in the post-COVID era that may inform public health policies.

## 5. Conclusions

More favourable prevalence estimates for physical activity and screen-time compliance and mental health symptoms were observed among Portuguese youth in 2024 than in 2022, while sleep guideline compliance did not differ significantly. These findings are based on independent cross-sectional samples and do not establish individual recovery from pandemic-related disruptions. Adolescents who met a greater number of 24-h movement behaviour recommendations had lower odds of depression, anxiety and stress symptoms. These findings suggest that school- and community-based strategies that simultaneously promote physical activity, healthy sleep, and balanced screen use warrant further evaluation. Longitudinal and experimental studies are needed to establish temporal ordering, assess causality and evaluate the long-term effects of integrated behaviour interventions on adolescent mental health.

## Figures and Tables

**Table 1:** Sociodemographic characteristics of the study participants by year and by sex.

	n (%) or Mean ± SD				
	Total (n = 5156)	2022 (n = 2775)	2024 (n = 2381)	Boys (n = 2478)	Girls (n = 2678)
**Age**	14.18 ± 2.23	14.54 ± 1.88	13.76 ± 2.52	14.19 ± 2.25	14.17 ± 2.22
**Grade**					
5th	254 (4.9)	0 (0)	254 (10.7)	116 (4.7)	138 (5.2)
6th	312 (6.1)	0 (0)	312 (13.1)	153 (6.2)	159 (5.9)
7th	716 (13.9)	400 (14.4)	316 (13.3)	366 (14.8)	350 (13.1)
8th	746 (14.5)	451 (16.3)	295 (12.4)	342 (13.8)	404 (15.1)
9th	797 (15.5)	455 (16.4)	342 (14.4)	394 (15.9)	403 (15.0)
10th	651 (12.6)	389 (14.0)	262 (11.0)	328 (13.2)	323 (12.1)
11th	955 (18.5)	631 (22.7)	324 (13.6)	451 (18.2)	504 (18.8)
12th	725 (14.1)	449 (16.2)	276 (11.6)	328 (13.2)	397 (14.8)
**School social support tiers**					
A	745 (14.4)	403 (14.5)	342 (14.4)	383 (15.5)	362 (13.5)
B	937 (18.2)	509 (18.3)	428 (18.0)	464 (18.7)	473 (17.7)
C	471 (9.1)	269 (9.7)	202 (8.5)	194 (7.8)	277 (10.3)
Not having support	3003 (58.2)	1594 (57.4)	1409 (59.2)	1437 (58.0)	1566 (58.5)

Abbreviations: SD, standard deviation. School social support in Portugal is determined by the family’s income bracket for child benefits, mainly based on household income and size. There are three main tiers (A, B, C), with tier A for the lowest-income families.

**Table 2:** Comparison of movement behaviours and DASS subscales between 2022 and 2024 among Portuguese children and adolescents, stratified by sex.

	n (%)								
	Total (n = 5156)			Boys (n = 2478)			Girls (n = 2678)		
	2022	2024	*p*	2022	2024	*p*	2022	2024	*p*
**Physical activity**			<0.001			<0.001			<0.001
Not attaining	2567 (92.5)	2055 (86.3)		1160 (88.4)	962 (82.5)		1407 (96.2)	1093 (90.0)	
Attaining	208 (7.5)	326 (13.7)		152 (11.6)	204 (17.5)		56 (3.8)	122 (10.0)	
**Screen time**			<0.001			<0.001			<0.001
Not attaining	2421 (87.2)	1710 (71.8)		1138 (86.7)	834 (71.5)		1283 (87.7)	876 (72.1)	
Attaining	354 (12.8)	671 (28.2)		174 (13.3)	332 (28.5)		180 (12.3)	339 (27.9)	
**Sleep**			0.365			0.551			0.466
Not attaining	1598 (57.6)	1401 (58.8)		716 (54.6)	654 (56.1)		882 (60.3)	747 (61.5)	
Attaining	1177 (42.4)	980 (41.2)		596 (45.4)	512 (43.9)		581 (39.7)	468 (38.5)	
**24-h MB attained**			<0.001			<0.001			<0.001
0	1327 (47.8)	927 (38.9)		567 (43.2)	415 (35.6)		760 (51.9)	512 (42.1)	
1	1185 (42.7)	976 (41.0)		591 (45.0)	481 (41.3)		594 (40.6)	495 (40.7)	
2	235 (8.5)	433 (18.2)		131 (10.0)	243 (20.8)		104 (7.1)	190 (15.6)	
3	28 (1.0)	45 (1.9)		23 (1.8)	27 (2.3)		5 (0.3)	18 (1.5)	
**DASS depression**			<0.001			0.136			<0.001
Normal	1556 (56.1)	1566 (65.8)		886 (67.5)	830 (71.2)		670 (45.8)	736 (60.6)	
Mild	356 (12.8)	237 (10.0)		148 (11.3)	101 (8.7)		208 (14.3)	136 (11.2)	
Moderate	453 (16.3)	335 (14.1)		165 (12.6)	132 (11.3)		288 (19.7)	203 (16.7)	
Severe	202 (7.3)	120 (5.0)		56 (4.3)	45 (3.9)		146 (10.0)	75 (6.2)	
Extremely severe	208 (7.5)	123 (5.2)		57 (4.3)	58 (5.0)		151 (10.3)	65 (5.3)	
**DASS anxiety**			0.001			0.454			<0.001
Normal	1577 (56.8)	1473 (61.9)		918 (70.0)	822 (70.5)		659 (45.0)	651 (53.6)	
Mild	222 (8.0)	177 (7.4)		100 (7.6)	73 (6.3)		122 (8.3)	104 (8.6)	
Moderate	425 (15.3)	351 (14.7)		171 (13.0)	143 (12.3)		254 (17.4)	208 (17.1)	
Severe	180 (6.5)	133 (5.6)		49 (3.7)	48 (4.1)		131 (9.0)	85 (7.0)	
Extremely severe	371 (13.4)	247 (10.4)		74 (5.6)	80 (6.9)		297 (20.3)	167 (13.7)	
**DASS stress**			<0.001			0.787			<0.001
Normal	1915 (69.0)	1819 (76.4)		1066 (81.3)	959 (82.2)		849 (58.0)	860 (70.8)	
Mild	286 (10.3)	193 (8.1)		90 (6.9)	78 (6.7)		196 (13.4)	115 (9.5)	
Moderate	284 (10.2)	209 (8.8)		83 (6.3)	65 (5.6)		201 (13.7)	144 (11.9)	
Severe	204 (7.4)	120 (5.0)		54 (4.1)	52 (4.5)		150 (10.3)	68 (5.6)	
Extremely severe	86 (3.1)	40 (1.7)		19 (1.4)	12 (1.0)		67 (4.6)	28 (2.3)	

Abbreviations: DASS, Depression Anxiety Stress Scales; MB, movement behaviours.

**Table 3:** Adjusted associations between movement guideline compliance and depression, anxiety and stress symptoms in the pooled sample.

	Odds Ratio (95% Confidence Interval)		
	DASS Depression (Mild to Extremely Severe)	DASS Anxiety (Mild to Extremely Severe)	DASS Stress (Mild to Extremely Severe)
**Physical activity**			
Not attaining	1.00 (ref)	1.00 (ref)	1.00 (ref)
Attaining	0.94 (0.77, 1.15)	1.04 (0.85, 1.26)	0.89 (0.71, 1.13)
**Screen time**			
Not attaining	1.00 (ref)	1.00 (ref)	1.00 (ref)
Attaining	0.69 (0.59, 0.81)	0.67 (0.57, 0.78)	0.63 (0.53, 0.76)
**Sleep**			
Not attaining	1.00 (ref)	1.00 (ref)	1.00 (ref)
Attaining	0.52 (0.46, 0.59)	0.57 (0.51, 0.65)	0.53 (0.46, 0.61)
**24-h MB attained**			
0	1.00 (ref)	1.00 (ref)	1.00 (ref)
1	0.57 (0.51, 0.65)	0.59 (0.52, 0.67)	0.61 (0.53, 0.70)
2	0.41 (0.33, 0.50)	0.49 (0.40, 0.59)	0.38 (0.30, 0.48)
3	0.41 (0.23, 0.71)	0.38 (0.21, 0.66)	0.22 (0.09, 0.51)

Abbreviations: DASS, Depression Anxiety Stress Scales; MB, movement behaviours. Adjusted for survey year, sex, age, grade, school social support and movement behaviours.

## Data Availability

The raw data supporting the conclusions of this article will be made available by the authors on request.
